# Reproductive site selection in a bromeliad breeding treefrog suggests complex evolutionary trade-offs

**DOI:** 10.1371/journal.pone.0207131

**Published:** 2018-12-05

**Authors:** Amanda Santiago Ferreira Lantyer-Silva, Anthony Waldron, Juliana Zina, Mirco Solé

**Affiliations:** 1 Department of Zoology, Herpetology Lab, Universidade Estadual Paulista “Júlio de Mesquita Filho”, Rio Claro, São Paulo, Brazil; 2 Departamento de Ciências Biológicas, Universidade Estadual de Santa Cruz, Ilhéus, Bahia, Brazil; 3 Departamento de Ciências Biológicas, Laboratório de Vertebrados, Universidade Estadual do Sudoeste da Bahia, Jequié, Bahia, Brazil; Universitat Trier, GERMANY

## Abstract

Reproductive site selection is a key determinant of fitness in many taxa. However, if the site characteristics that enhance offspring survival are detrimental to the parent’s survival or mating success, then complex evolutionary trade-offs occur. In the Brazilian Atlantic Forest, males of the treefrog species *Aparasphenodon arapapa* use the temporary water bodies in forest-floor bromeliads to court and mate. Males fit tightly into the plant with the head blocking the access and after mating, stay in the bromeliad with the offspring. Since evaporation of the temporary water body inside the bromeliad results in reproductive failure, we expected that males would simply choose the largest bromeliad tanks with the most water. We found that although this was generally true, males seemed to avoid both very large bromeliads and very high water volumes. Field observations suggested a trade-off mechanism for this pattern, whereby very large and water-filled tanks would reduce the male’s ability to effectively seal the tank entrance, avoid predation, or call to mating females. Males also avoided bromeliads with leaf litter and preferred slightly inclined plants. Our results indicate that during reproductive site selection, this bromeliad-breeder needs to engage in complex trade-offs between selection pressures, balancing water requirements against the need for defense and potentially, the ability to attract a mate.

## Introduction

Reproductive site selection is a key factor underpinning organismal fitness [1]. However, the factors that define a reproductive site’s suitability are multiple. For parental and offspring fitness, site qualities such as resource availability, predation risk and competition are key considerations [2,3,4,5]. For mating success, individuals (usually males) will also need to choose sites that attract a mate, particularly when courtship and reproduction occur in the same place [6,7,8,9]. With so many considerations in play, sites could sometimes be highly suitable for one sub-need but far less suitable for another, suggesting a need for a trade off calculus. For example, Arabian Desert Hoopoe Larks (*Alaemon alaudipes*) prefer to nest and incubate in open areas where they can detect approaching predators, but open areas also offer less shelter and in the hottest parts of the year, more enclosed areas therefore become preferable [10].

Evolutionary trade offs in other areas have been widely studied [11,12,13], including the trade offs involved in foraging under predation risk [14,15,16], which has a clear parallel to reproducing under predation risk. To date, however, knowledge and investigation of trade offs in reproductive site selection remains very limited. One reason may be that trade offs are subtle and difficult to detect in common situations. Potentially, some of the best opportunities to study trade offs arise when reproductive strategies are extreme and multifaceted, generating extensive variation in needs and powerful consequences of mistakes in site choice [17]. One of the most challenging and multifaceted reproductive strategies is that of anurans adapted to reproduce in temporary, water-filled plant cavities such as bromeliads, tree holes, nut capsules and flower bracts [18,19]. Such cavities are known technically as “phytotelmata” (greek, *phyto* = plant, *telma* = pond) [17,18]. The tadpole stage of anurans is typically dependent upon water, and so the use of phytotelmata already represents a broad compromise between an increased risk of desiccation and a decrease in predation and competition [18,19,20,21,22]. However, the precise balance of risk and reward will be different across individual phytotelmata, and it is these differences that will drive selection of the precise reproductive site from among the set of available phytotelms. Differences in water quantity and quality, plant architecture and plant positioning in the environment have all been studied as individual drivers of site choice [20,21,22]. Here, we extend this classic approach of studying individual preference drivers independently, to explore how combinations of plant characteristics drive site choice and in particular, how contradictory choice patterns indicate likely trade offs between different aspects of fitness.

As our study system, we use Bahia’s Broad-snout casque-headed Tree Frog *Aparasphenodon arapapa*, a range-restricted anuran of the Brazilian Atlantic rainforest that has an extraordinarily complex set of phytotelm-dependent behaviors [23,24]. Males select a bromeliad, insert themselves into the water-containing central tank, and then attempt to court females by extruding the head above water and calling. If a female accepts the courtship, then mating, egg laying and fertilization all occur within the same bromeliad. The male then remains in the central tank for at least the three weeks that the larvae need to develop [24]. Additionally, the male treefrog exhibits an adaptive behavior known as “phragmosis” [25,26], in which it sits in the base of the bromeliad tank and blocks the entrance by tilting its head to form a seal. The cranium is hyperossified and phragmosis seems likely to be an anti-predator adaptation, although it remains unclear whether it is the male, or the offspring, or both that benefit [25,26]. Phragmosis may also defend against invasion of the reproductive territory (the bromeliad) by competitors. The larvae feed on conspecific eggs laid by the female (oophagy) and thus, the larval food resource is scarce and potentially requires protection as well.

In summary, the net suitability of each bromeliad is a complex function of its suitability for calling and attracting a female; its suitability for raising the young in a high-risk environment where the key resource of water can disappear; and its suitability for the suite of anti-predator behaviors displayed, some of which may also serve to exclude competitors. All these behaviors have important fitness consequences that could contradict each other. We would expect the primary preference of a searching *A*. *arapapa* individual to be for high water content and thus, for large bromeliads (since plant size is the first external cue of possible water content). However, the behaviors of phragmosis and extruding the head to call would make very large tanks or very deep water relatively unattractive, trading off against this primary focus. Statistically, the hypothesized trade off would cause the preference functions to interact with each other in potentially non-linear ways, and so we tested for such a pattern. Other factors may also alter the primary attractiveness of large or water-filled bromeliads, such as water quality or ease of access to the bromeliad tank [21,27]. We therefore additionally analyzed the importance of such factors, using pH [21] and the level of debris in the water as aspects of water quality and bromeliad inclination as a measure of accessibility.

## Material and methods

### Ethics statement

The study was approved by the Ethics Committee on Animal Use of the Universidade Estadual de Santa Cruz (Ilhéus, Bahia, Brazil) (CEUA UESC 002/12). Toe-clipping was made using lidocaine as local anesthesia. National legal authorization was provided by Instituto Chico Mendes de Conservação da Biodiversidade (ICMBio) to MS (13708–1).

### Field data sampling

We conducted the study at Reserve Boa União (15º04′ S; 39º03′ W, 95 m asl, 112 ha), located in the municipality of Ilhéus, state of Bahia in Brazil. This is an area of tropical and humid climate characterized by a mosaic of shrubby and arboreal vegetation inside patches of sandy soil surrounded by forests, similar to an arboreal ‘restinga’ [28,29].

To carry out the study, we identified a bromeliad-dense area lying along approximately 400m of a central trail of the reserve. Within that area, we randomly selected a sampling area of 80m x 350m. We defined two classes of bromeliad: those being used as reproductive sites, and those not being used. We used the presence of a calling male as an indicator of reproductive site use. Thus, bromeliads occupied by a calling male (occupied by male or “OBM” bromeliads) were classified as sites in reproductive use, and bromeliads not occupied by a male (“NOBM” bromeliads) were classified as sites not in reproductive use. We acknowledge that some NOBM cases may potentially have been used on days when we did not sample, but assume that on average, over multiple surveys, the plants where we found males represented greater site suitability than the plants where we never found males, as is common in habitat selection studies [30,31].

To collect data on NOBM bromeliads, we created 24 circular plots of 2m radius each within our study area, using stratified random sampling (each plot had between five and seven bromeliads). On approximately weekly site visits from November 2011 to March 2012, we identified all NOBM bromeliads within each plot (n = 169) and took measurements of bromeliad tank size (depth and diameter), tank water volume, pH and level of debris (leaf litter), and bromeliad inclination (Table 1). We measured depth as the maximum depth of the central bromeliad tank and diameter at the top of the tank, taking the shortest distance across the top of the leaf basket. We measured water volume by aspiring water from bromeliad central tank into a measuring cylinder and rounding volume to the nearest 1.0 ml. We measured leaf litter as an ordinal variable (from 0 to 3) defined as follows: 0- no litter, 1- small amount of litter, 2- large amount of litter but not enough to prevent an individual of *A*. *arapapa* from passing into the bromeliad central tank; 3- very large amount of litter, fully obstructing passage into the tank [27]. We estimated inclination of the bromeliad using a three-point scale: 0- upright plants, 1- slightly inclined, 2- heavily inclined (close to the ground). We measured the pH of the water in the bromeliad tank using a water quality sensor Sanxin Sx-620 with a precision of 0.1.

**Table 1:** Characteristics of bromeliads with *Aparasphenodon arapapa* males (occupied bromeliads) and without (not occupied bromeliads). Summary statistics shown use the full dataset of occupied bromeliads, without removing information of bromeliads with recaptured males. SD = standard deviation, n = number of bromeliads sampled.

	Occupied bromeliads by males				Not occupied bromeliads by males			
Variables	mean	SD	range	n	mean	SD	range	n
**Diameter (cm)**	2.7	0.8	0.8–5	74	2.8	1.0	0.8–7.5	165
**Tank depth (cm)**	17.9	4.7	9–30	74	14.6	6.9	6–8	165
**Water volume (ml)**	21.4	11.3	7–60	74	7.0	10.8	0–80	165
**pH**	4.3	0.8	3–6.99	57	4.3	0.6	3.3–5.8	67

To collect the same data for OBM bromeliads, we identified all bromeliads (n = 105) we found occupied by males over a total of 43 visit nights (from 1800h to 2400h each visit between November 2011 and October 2012), carefully dislodging each male and taking the same measurements of plant characteristics as we had for NOBM bromeliads, then releasing the male back into the same bromeliad where it was found. (We extended the OBM surveying period up to October to collect sufficient OBM data; earlier field observations (confirmed during the current study) had indicated that reproduction in *A*. *arapapa* was not seasonal but largely continued throughout the year). We measured the snout to vent length of all males found in a bromeliad, for comparison with the depth of the bromeliad tank in which the male was found. We also toe-clipped males [32] and checked for cases where a male had used more than one bromeliad over our study period. Re-use may occur because some of the characteristics of the bromeliad are dynamic e.g. the water volume and pH can change depending on rainfall. However, we emphasize that the volume and pH of OBM and NOBM bromeliads always reflected the night on which they were found occupied or unoccupied, and so the site preference observed would be a response to the dynamic site conditions at the moment of observation.

### Data analysis

We first visually examined differences between OBM and NOBM bromeliads by plotting the trait space for each class. We then statistically modeled the probability of a bromeliad being occupied, using the predictor variables in Table 1 and taking an information-theoretic approach in which all models with delta AICc <2 are defined as the “best fitting model set” [33]. Tank diameter and depth were square root transformed and water volume was cube root transformed. It was not clear a *priori* whether the unit distances between ordinal values of inclination and litter (e.g. 0,1,2,3) represented a scaling of effects biologically appropriate to the study organism, so we ran two sets of models that treated these ordinal variables in two different ways: first, as continuous variables (which assumes approximate biological meaning in the ordinal scale), and second, as categorical factors (which drops this assumption). All non-categorical variables were centered and standardized by a (x-mean(x))/sd(x)) transformation. Our trade-off hypotheses envisaged that water volume and bromeliad size may have contradictory influences on site preferences, so we included candidate models that tested for interactions between volume and tank depth, and between volume and tank width. Early exploration of the data suggested a strong non-linear response to water volume. We therefore also tested Generalized Additive Models (GAMs) with binomial errors and a logit link [34] and also Generalized Linear Models (GLMs) using a quadratic term for volume.

We excluded a number of observations prior to carrying out final model selection. For the OBM part of the dataset, we excluded one record where the male was found in the lateral axils of the plant rather than in the central tank, since the physical characteristics of central and lateral axils are not comparable. In a further 30/105 OBM cases (29%), we found a male that had been previously recorded in a different OBM bromeliad, including one male that had used three bromeliads. Analyzing the same male more than once may introduce biases, so we analyzed the first bromeliad in which each male was found. We then repeated the analysis using the last bromeliad in which the male was found, and compared the results to see whether they were sensitive to this choice. For the NOBM part of the dataset, pH could not be measured in bromeliads containing no water (generating a missing variable). However, excluding all water-free bromeliads would have removed a large and highly non-random part of the sample and at the same time, models that included pH consistently had extremely poor information-theoretic support. We therefore ran a final set of analyses that retained bromeliads with no pH measurement (removing pH from the candidate model set) but still excluded bromeliads with missing data (n = 4) for any other candidate variable. With these exclusions, our final sample size was 74 occupied bromeliads and 165 not occupied bromeliads (S1 Table).

To explore potential multicollinearity effects, we calculated VIF (Variance Inflation Factor) scores on all the terms identified in all models with AICc<2.0, where scores of >5 may indicate that the regression coefficient estimative is affected by collinearity [35] and also cross-correlations between all variables (S2 Table). All analyses were carried out using the R statistical package version 3.0.2 [36], with package MuMIn for model selection [37], and package mgcv for GAMs [38,39].

## Results

**Fig 1:**
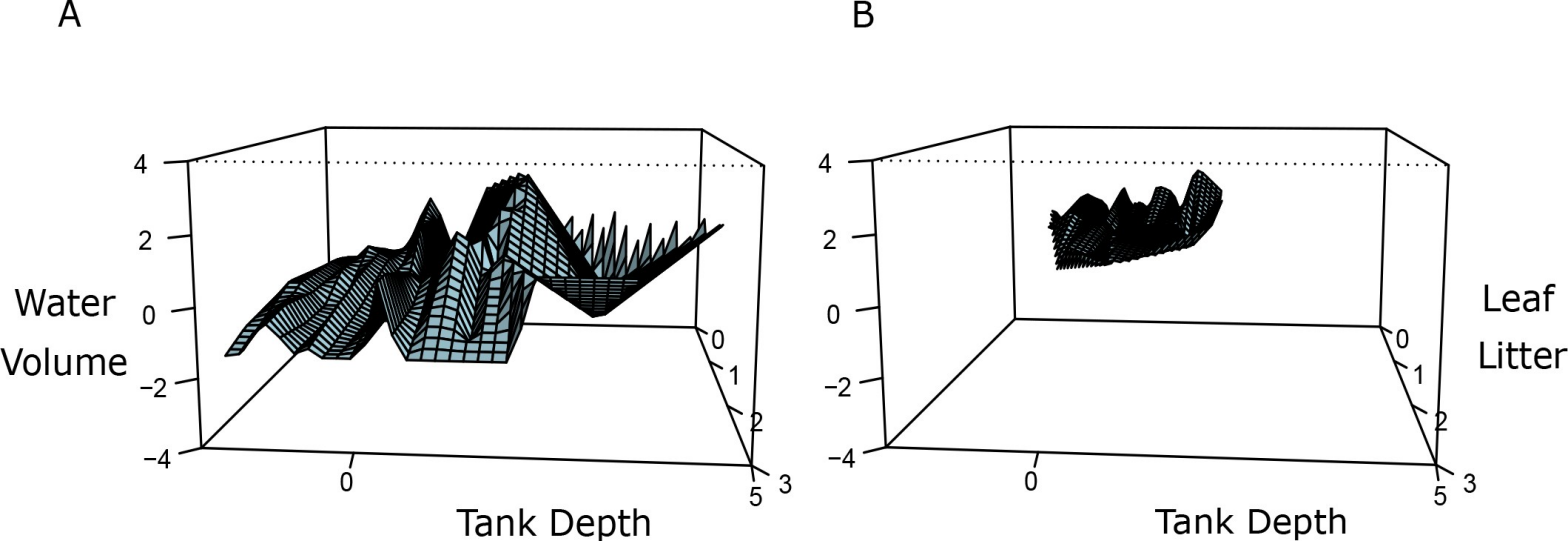
Trait space showing differences between data on bromeliads not occupied by a male of *Aparasphenodon arapapa* (NOBM) and occupied ones (OBM). (a) Unoccupied bromeliads in three-dimensional trait space, where the dimensions are bromeliad tank depth, water volume, and leaf litter. (b) Occupied bromeliads in the same three-dimensional trait space.

**Fig 2:**
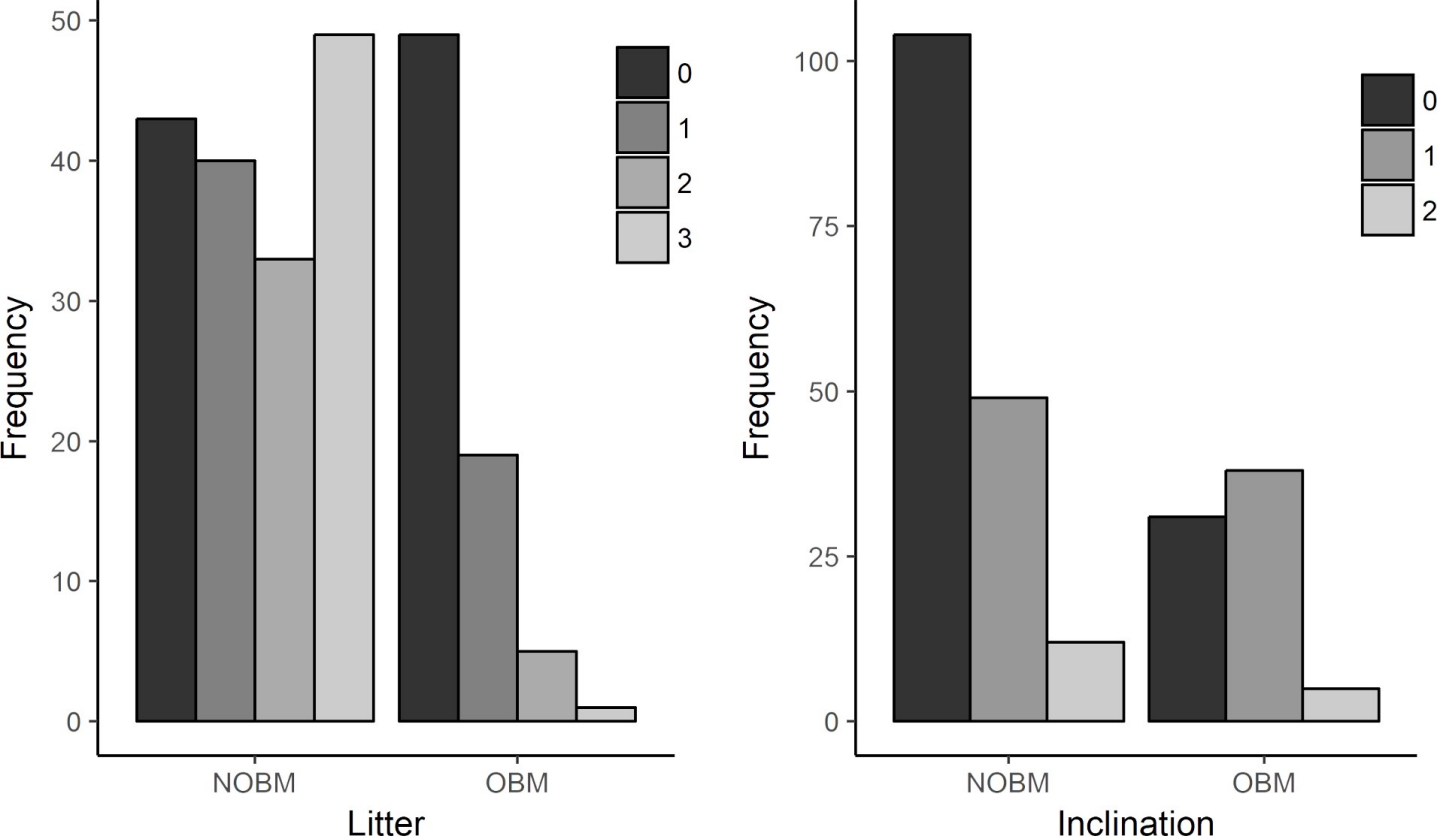
Differences between data on bromeliads not occupied by a male (NOBM) and occupied ones (OBM). (a) Differences in leaf litter. (b) Differences in inclination. Inset of figures show categories. Litter/ inclination: 0 = no litter/upright plants; 1 = small amount of litter/slightly inclined plants; 2 = large amount of litter but not enough to prevent an individual of the anuran from passing through the central tank/ heavily inclined; 3 = very large amount of litter with no passage free through central tank/ it is not a category to “inclination”.

Visual comparison and summary statistics suggested that bromeliad trait space for reproductive sites was a clearly distinct subset of the total trait space available (Figs 1 and 2, Table 1). Compared to NOBM bromeliads, OBM bromeliads generally had higher water volumes, deeper tanks, less leaf litter (Figs 1 and 2, Table 1, S1 Table), and slightly more inclination (Fig 2, S1 Table). Nevertheless, the very largest bromeliad tanks were never found in reproductive use (Fig 1), even though these very large tanks generally held high some of the highest volumes of water (S3 Table, S4 Table). There were no clear visual differences between the pH and tank diameter values of OBM and NOBM bromeliads.

**Fig 3:**
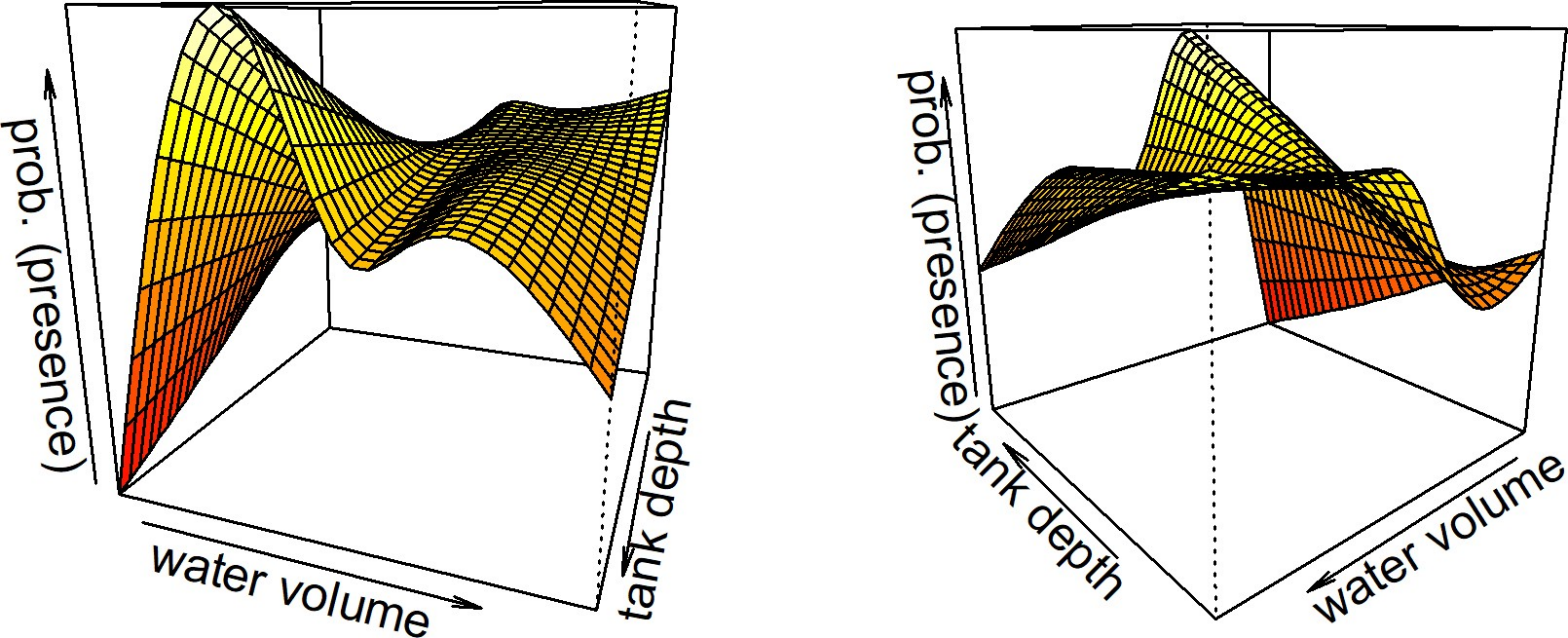
Tank depth and water volume interact non-linearly in the statistical model of reproductive site choice (“prob (presence)”). The non-linear two-factor response surface is shown from two different angles (a,b). Note how the general increase in preference with phytotelm size (bromeliad tank depth) is reversed at very low and very high water volumes, and the general increase in preference with water volume is reversed in very deep bromeliads.

When we tested statistical models of reproductive site preferences, two best-fit models emerged (Table 2, S2 Table). The model with the lowest AICc generally supported our visual observations that site occupancy probability increased with tank depth (Fig 3, Table 3). However, if bromeliads had a very high water volume (>77^th^ percentile of volume), the pattern was reversed i.e. site occupancy probability started to decrease with tank depth in the most water-filled bromeliads (Fig 2, S1 Fig, S2 Fig). Similarly, occupancy probability generally increased as water volume increased (with an asymptotic limit) (S2 Fig). However, in the deepest tanks, site preference reversed this general pattern and decreased with increasing water volume (Fig 2, S2 Fig). Site occupation probability decreased as litter levels increased and was significantly greater for inclination = 1 than for inclination = 0 (Table 3). The model with the second lowest AICc contained similar terms and effect directions, but also added a term for tank diameter, with occupancy probability increasing as tank diameter decreased (S2 Table).

**Table 2:** AICc table for all models with delta AICc<10. Vol = volume; diam = diameter; incl = inclination (factor); spline = non-linear cubic spline fitted; 2spline = interacting cubic splines fitted to both terms. In parametric models, standardized coefficients rounded to 2 decimal places are shown. NA means not included in the model. “+” indicates that the term was modelled with a smoother. See S3 Table for all models.

**AICc**	131.25	131.95	138.20	138.96
**delta AICc**	0.00	0.70	6.95	7.71
**model weight**	0.56	0.40	0.02	0.01
**water vol.**	spline	spline	spline	3.72
**tank depth : water vol.**	2spline	2spline	NA	NA
**tank depth**	NA	NA	spline	NA
**tank depth : tank diam.**	NA	NA	2spline	NA
**Incl. (f)**	+	+	+	+
**tank diam.**	NA	-0.33	NA	0.36
**leaf litter**	-1.17	-1.06	-1.22	-1.09
**water vol.^2**	NA	NA	NA	-1.44
**tank diam. : water vol.**	NA	NA	NA	-0.98

**Table 3:** The best-fitting model (lowest AICc) explaining reproductive site selection. Adjusted R^2^ = 0.66. Spline = cubic spline fitted, 2spline = cubic spline fitted to both terms simultaneously (i.e. a non-linear interaction). Z-values are shown for parametric terms and Chi-squared values for fitted splines. All variables are z-standardized. x = interaction. NA = not transformed in the model.

Regression term	Coefficient	z-value/chi sq.	p-value
**Volume**	spline	38.09	<0.0001
**Tank depth x volume**	2spline	12.98	0.016
**Litter level**	-1.17	-3.07	0.0002
**f(Inclination = 1)**	1.71	NA	0.001
**f(Inclination = 2)**	1.10	NA	0.29

Correlations between the terms were generally moderate (S3 Table). The strongest correlations were a positive association between tank depth and water volume (Pearson’s r = 0.447), a negative association between leaf litter and water (Pearson’s r = – 0.317), and a positive association between pH and water volume i.e. more water-filled tanks were less acid (Pearson’s r = 0.574). However, all VIF values were <1.55 in the best-fitting model set. The depth of the bromeliad tanks occupied by males was a median 3.74 times larger than the body-length (snout to vent length) of the occupying male. For the 30 cases where a male had used more than one bromeliad, there was no substantive difference in the results for models using the first bromeliad recorded and models using the last bromeliad recorded.

## Discussion

Our results show that the bromeliads chosen as reproductive sites are a highly non-random subset of the available bromeliads, implying that site selection is indeed occurring and that a set of preferences for bromeliad traits underlies site choice behavior. Some of the characteristics that we found to drive site preferences would be visible to a searching male on the forest floor (e.g. the size and inclination of the bromeliad), whereas others would only become visible once a male had climbed up into the bromeliad (the water volume and leaf litter level). Our results also showed that this climb would require the male to ascend approximately four times its own body-length up the side of the bromeliad, before being able to directly determine the non-visible bromeliad characteristics. Performing such a climb repeatedly, in the search for an appropriate site, seems likely to represent an expenditure of energy large enough to impact upon fitness.

*A*. *arapapa* has an aquatic larval phase entirely associated with the ephemeral water content of a bromeliad. Given the primary importance of the water body persisting long enough to allow the tadpoles to develop, our *a priori* expectation was that site preference would increase with water volume [40]. Since water volume would not be immediately visible to the male on the forest floor, we also expected that preference would increase with tank depth or tank diameter, both of which would be obvious external proxy cues for the likely water content inside. Our results partially confirmed these simple expectations, suggesting that in general, site preference indeed increases with water volume and with the depth of the bromeliad tank. However, our interaction results also showed that in some cases, male preference patterns were the opposite of these simple expectations. Males avoided bromeliads that had both very deep tanks and very high volumes of water. In our second best-fitting model (i.e. second-lowest AICc value), male site preference also decreased with tank diameter, the opposite of the pattern for tank depth (Table 2).

These complex and often contradictory results suggest that a powerful set of trade-offs is operating, driven by selective pressures on different aspects of reproductive site preference. We suggest that the unexpected avoidance of very large bromeliad tanks with very large water volumes, plus the surprising finding that males prefer small-diameter tank entrances, may be due to a trade-off between the importance of water and the importance of defending against predation (and/or competition) through phragmosis—the behavior where males seal the tank entrance with their fortified head [24,26]. As observed in the field, phragmotic behavior in *A*. *arapapa* requires the male to shrink down into the base of a tapered tank and indeed, we often found males so tightly fitted into the bromeliads that considerable leverage with a spatula was needed to dislodge them. A very large tank is likely to hinder the male's ability to shrink down tightly enough or seal the entrance comprehensively enough. The need for a tight seal would also explain why site preference was unexpectedly higher for smaller tank diameters than for larger ones. The apparent behavioral aversion to high water volumes in combination with a large tank may also follow from such a mechanism: very deep water would push the frog's body upwards, again making it more difficult to fit tightly into the tapered tank bottom. There are other hypothetical mechanisms that could also contribute to a trade-off pattern (potentially in combination with a phragmosis explanation). The male has to extrude his head out of water to perform courtship vocalizations, which is essentially achieved by placing the find feet on the base of the bromeliad tank. Very deep water would hinder this behavior (and hence reproductive fitness), especially if the water was so deep that the individual's head became positioned some distance beneath the surface. Thus, both the need to avoid predation and/or competition, and the need to attract a mate, could potentially be conflicting with the need to place the tadpoles in a large enough ephemeral water body, generating trade-off patterns.

Our results also show that preference decreased as the quantity of leaf litter in the bromeliad increased, corroborating what was found for another bromeligenous anuran from the Atlantic Forest in Brazil, *Phyllodytes melanomystax* [41]. The simplest mechanism explaining this observation is that excessive leaf litter impedes access to the tank. Indeed, the importance of tank access was also suggested by our finding that males prefer inclined bromeliads, which would presumably be easier to climb into (Table 2, Fig 1). High litter levels were also correlated with low water volume (Pearson’s r = -0.31, see Results). The avoidance of litter may therefore be partly a function of the preference for water, with leaf litter taking up space that could otherwise hold water.

We found no evidence that pH affected site choice in *A*. *arapapa*, and similarly negative results for pH have been found for other anuran species (e.g. *Phyllodytes luteolus* [20,42] and *P*. *melanomystax* [41]). However, we note that pH has been found to influence site choice in certain other anurans (e.g. *Lithobates sylvaticus* and *Ambystoma maculatum* [43] and *Scinax perpusillus* [21]). We suggest that further research is therefore needed on water chemistry effects. One interesting observation in the current study was that acidity decreased with water volume (Pearson’s r = 0.574, see Results). In such species where pH does have an effect on tadpole survival and growth [21,44], it may therefore be appropriate to extend our trade-off framework, to study the interacting influences of water volume and acidity on site choice.

In conclusion, the multiple uses of the bromeliad by *A*. *arapapa* create a study system where multiple evolutionary trade-offs can be explored. We have focused on the natural selection aspects of fitness but there are interesting possibilities to extend a trade-off framework to other types of selection. For example, the need to extrude the head to call to females, even if this means rejecting the richest water resources, suggests a potential trade off between sexual selection and natural selection [45]. The situation could be even more complex if females are choosing males based on both their calls [46] and on the key site-quality trait of water. In such a case, the perceived attractiveness of the mate and the perceived attractiveness of the reproductive site containing the mate may also partially contradict. Such possibilities remain hypothetical until further research can be carried out.

Loss of reproductive sites is an important form of habitat loss, itself one of the key drivers of species decline [47]. The study of evolutionarily complex organisms such as bromeliad-breeding frogs is partly motivated by our desire to conserve the most extraordinary features of earth’s biodiversity and evolutionary processes, especially if they are rare [48]. Indeed, *Aparasphenodon arapapa* occupies a restricted range in one of the world’s most highly threatened ecosystem (the Brazilian Atlantic rainforest [49]). The management of reproductive sites is often a key component of such species conservation, but the site features studied (and therefore managed) are typically restricted to fairly straightforward set of offspring requirements [2]. Our analysis suggests that while research on straightforward needs is important, surprising and contradictory requirements can exist that analyses not considering trade-offs would miss. Indeed, the more complex the biological adaptation we are trying to preserve, the more likely it is that trade offs become an important factor in a species' evolutionary ecology and thus, its survival. If we wish to conserve some of the least common and most fascinating aspects of our natural heritage, future research may therefore need to consider and test for reproductive site trade-offs far more frequently.

## Supporting information

S1 Fig The influence of bromeliad tank depth on reproductive site preference at different water volumes. Panels show how the shape of the preference function for tank depth (i.e. probability of presence given tank depth) depends on the water volume in the tank: a = 25^ile^ of water volume, b = 50^ile^ (i.e. the median bromeliad water volume), c = 77^ile^, d = 90^ile^. Rug plots at bottom show the empirical distribution of tank depths.
(DOCX)

S2 Fig The influence of bromeliad water volume on reproductive site preference at different tank depths. (a) At the average (mean) bromeliad tank depth, male preference increases with water volume. (b) In bromeliads with a very deep tank, however (90^th^ percentile is shown), high volumes of water reduce site preference. Rug plots at bottom show the empirical distribution of water volumes.
(DOCX)

S1 Table Raw data. MinD = minimum diameter; MaxL = maximum central tank length, NA = data not available.
(TXT)

S2 Table AICc tables for all candidate models. (XLSX)

S3 Table Cross-correlations between explanatory variables. (XLSX)

S4 Table Raw data on frequency of bromeliads with *Aparasphenodon arapapa* males (used bromeliads) and without (not used bromeliads) according to the systematic sample. N values show the number of plants in each category. 0 = no litter/upright plants; 1 = small amount of litter/slightly inclined plants; 2 = large amount of litter but not enough to prevent an individual of the anuran from passing through the central tank/ heavily inclined; 3 = very large amount of litter with no passage free through central tank/ it is not a category to “inclination”.
(XLSX)
