# Supplementary material for: Reproductive site selection in a bromeliad breeding treefrog suggests complex evolutionary trade-offs
Source: PLoS One. 2018 Dec 5;13(12):e0207131. doi: 10.1371/journal.pone.0207131 (PMC6281282; doi:10.1371/journal.pone.0207131)
Supplement: S1 Fig — Panels show how the shape of the preference function for tank depth (i.e. probability of presence given tank depth) depends on the water volume in the tank: a = 25ile of water volume, b = 50ile (i.e. the median bromeliad water volume), c = 77ile, d = 90ile. Rug plots at bottom show the empirical distribution of tank depths. (DOCX) [file pone.0207131.s001.docx]

| Probability of presence |  |
| --- | --- |
|  | Bromeliad tank depth (z-transformed) |
|  |  |

Supplementary Figure 1. The influence of bromeliad tank depth on reproductive site preference at different water volumes. Panels show how the shape of the preference function for tank depth (i.e. probability of presence given tank depth) depends on the water volume in the tank: a=25^ile^ of water volume, b=50^ile^ (i.e. the median bromeliad water volume), c=77^ile^, d=90^ile^. Rug plots at bottom show the empirical distribution of tank depths.
