# Supplementary material for: Reproductive site selection in a bromeliad breeding treefrog suggests complex evolutionary trade-offs
Source: PLoS One. 2018 Dec 5;13(12):e0207131. doi: 10.1371/journal.pone.0207131 (PMC6281282; doi:10.1371/journal.pone.0207131)
Supplement: S2 Fig — (a) At the average (mean) bromeliad tank depth, male preference increases with water volume. (b) In bromeliads with a very deep tank, however (90th percentile is shown), high volumes of water reduce site preference. Rug plots at bottom show the empirical distribution of water volumes. (DOCX) [file pone.0207131.s002.docx]

Supplementary Figure 2. The influence of bromeliad water volume on reproductive site preference: (a) At the average (mean) bromeliad tank depth, male preference increases with water volume. (b) In bromeliads with a very deep tank, however (90^th^ percentile is shown), high volumes of water reduce site preference. Rug plots at bottom show the empirical distribution of water volumes.
